# Cationic microbubble loading hSIRT3 and hTIMP3 optimize cardiac-targeted delivery and myocardial protection in the porcine MI/R model

**DOI:** 10.1016/j.mtbio.2025.102234

**Published:** 2025-08-22

**Authors:** Peian Cai, Kegong Chen, Xionghai Qin, Xingpei Jiang, Xuan Jiao, Kexun Liu, Erliang Guo, Zipeng Li, Xianxin Qiu, Chang Liu, Lu Sun, Junbo Chuai, Jie Wu, Wei Chen, Hai Tian

**Affiliations:** a Department of Cardiovascular Surgery, The Second Affiliated Hospital of Harbin Medical University, Harbin, Heilongjiang province, China; b Future Medical Laboratory, The Second Affiliated Hospital of Harbin Medical University, Harbin, Heilongjiang province, China; c The Affiliated Cancer Hospital of Zhengzhou University & Henan Cancer Hospital, Zhengzhou, Henan Province, China; d Department of Thoracic Surgery, The First Affiliated Hospital of Anhui Medical University, Hefei, Anhui province, China; e Department of Thoracic Surgery, Harbin Medical University Affiliated Cancer Hospital, Harbin, Heilongjiang province, China; f Department of Cardiovascular Surgery, Peking University Shenzhen Hospital, Shenzhen, Guangdong province, China; g Key Laboratory of Preservation of Human Genetic Resources and Disease Control in China (Harbin Medical University), Ministry of Education; Department of Medical Genetics, School of Basic Medical Sciences, Harbin Medical University, Harbin, Heilongjiang Province, China

**Keywords:** Cationic microbubbles, Gene therapy, Myocardial ischemia-reperfusion injury, Porcine model, Dual-gene synergistic therapy

## Abstract

Myocardial ischemia-reperfusion (MI/R) injury limits the therapeutic effects of revascularization in acute myocardial infarction. In this study, we investigated whether human SIRT3 (hSIRT3) and TIMP3 (hTIMP3) could achieve targeted delivery with the assist of cationic microbubbles (CMBs) and a synergistic protection effect on porcine MI/R myocardium. Firstly, CMBs carrying the hSIRT3 or hTIMP3 plasmids were used individually or synergistically for cardiac-targeted delivery in MI/R pigs. After 7 days of observation, hSIRT3 and hTIMP3 were mainly enriched in myocardium, especially in the infarction center, without additional increase in cTNI and pathological damage to non-cardiac organs. At the same time, hSIRT3 and hTIMP3 exerted a protective role against myocardial injury, as gene therapy significantly inhibited myocardial apoptosis, inflammation and oxidative damage. After 90 days of observation, hSIRT3 and hTIMP3 application exerted an inhibiting effect on development of heart failure, as the strategy significantly increased the density of vascular, and limited the myocardial fibrosis, area scar size, the decline of cardiac function. As expected, collaborative applications of hSIRT3 and hTIMP3 showed a better protective effect than hSIRT3 or hTIMP3 application alone. Collectively, hSIRT3 and hTIMP3 delivered with CMBs in heart could exert positive effect on myocardial protection after MI/R in pigs.

## Graphical abstract

**Figure:**
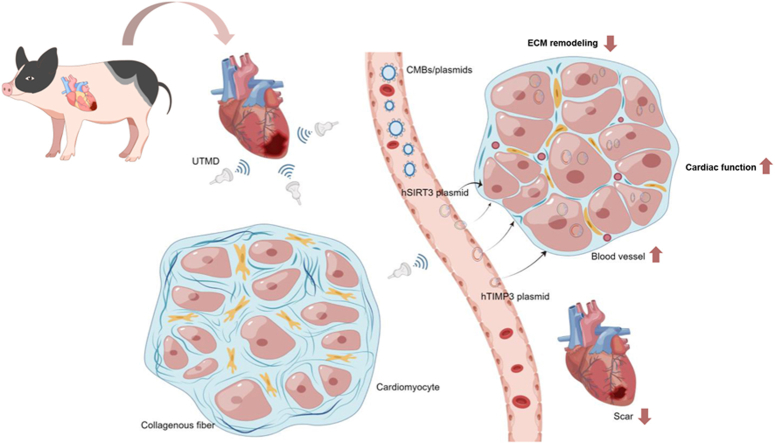

## 1 Introduction

Advancements in various strategies for reperfusion timing have greatly reduced the mortality of patients with acute myocardial infarction (MI). However, reperfusion injury caused by ischemic myocardium after restoring of blood supply diminishes the expected benefits and long-term effects of coronary revascularization [1,2]. Although many animal studies have been conducted on the pathogenesis and treatment of myocardial ischemia-reperfusion (MI/R) injury, some studies have not achieved encouraging results in relevant clinical trials, limiting its large-scale promotion and application [[3], [4], [5]].

Oxidative stress, inflammatory responses, mitochondrial dysfunction, and proteolytic signaling have long been recognized as the main pathological processes involved in MI/R injury [6]. Sirtuin 3 (SIRT3) is an NAD^+^-dependent deacetylase that plays a pivotal role in antioxidant and mitochondrial homeostasis [7]. Clinical studies have shown that the expression of SIRT3 decreases with age, consistent with an increase in the incidence of cardiovascular diseases [8]. Simultaneously, cardiovascular disease risk factors may lead to reduced SIRT3 level [8]. In animal studies, the absence of SIRT3 is associated with mitochondrial oxidative stress damage, vascular inflammation injury and a decrease in manganese superoxide dismutase (MnSOD) activity in myocardial tissue [9,10]. SIRT3 can relieve myocardial damage post-MI by inhibiting mitochondrial fission [11], whereas overexpression of SIRT3 can delay the progression of heart failure (HF) by improving mitochondrial biological function [12].

MI/R injury involves dynamic changes after the initial pathological process, and adverse extracellular matrix (ECM) remodeling is a key factor leading to ventricular dysfunction and HF after MI/R injury [13]. Adverse ECM remodeling is mainly result from the unbalance between tissue inhibitors of metalloproteinases (TIMPs) and matrix metalloproteinases (MMPs) expressions [14]. In the members of TIMPs family, TIMP3 is abundantly expressed in the heart [15]. In clinical observational studies, a decrease in TIMP3 and an increase in MMPs have been detected in the serum of patients with MI during hospitalization [16]. And the absence of Timp3 in mice could accelerate post-MI ventricular remodeling and fibrosis [17]. Local injection of Timp3 into the infarcted myocardium of mice inhibited adverse remodeling of the heart after MI [18]. The use of tissue engineering technology to attach recombinant TIMP3 protein to the surface of the porcine infarcted myocardium has been reported to effectively reduce infarct size, inhibit myocardial fibrosis and ventricular remodeling, and improve cardiac function [19].

Therefore, the administration of exogenous SIRT3 and TIMP3 during myocardial injury may be an advantageous strategy to ameliorate adverse long-term outcomes after revascularization post-MI. Cationic microbubbles (CMBs) have been demonstrated its ideal gene-carrying capabilities and could achieve minimally invasive targeted gene delivery with the assistance of ultrasound-targeted microbubble destruction (UTMD), making it highly promising for clinical application [20]. CMBs can be electrically coupled with anionic DNA, which not only protects DNA from premature degradation by nucleases in the circulatory system, but also enables the release of a large amount of DNA and gene transfer in a focal area once sonoporation is induced, achieving the goal of targeted gene therapy [21]. Gene therapy mediated by CMBs has shown optimal therapeutic effects in rodent models of cardiovascular diseases [[21], [22], [23], [24]]. Moreover, CMBs can also achieve cardiac-targeted gene delivery in pigs. A previous study used UTMD to locally deliver the microRNA-21 bound to CMBs into normal myocardia and observed it for four-days. The microRNA-21 was successfully expressed in heart tissue, and myocardial troponin was not significantly increased during the observation period, nor did it cause morphological tissue damage [25]. In addition, after transfecting the adeno-associated virus vectors carrying luciferase reporter gene on CMBs via UTMD into healthy porcine heart, luciferase was reported to maintain significant activity in cardiac tissue, while only very weak luciferase activity was observed in non-cardiac tissue during an observation period of one month [26]. These results indicate that CMBs assisted by UTMD can achieve targeted gene transfection in myocardium of large animals, like pigs. Due to the close proximity of pigs to humans in terms of cardiovascular pathophysiology, structural function, and anatomy [27,28], theoretically, CMBs can also introduce exogenous genes into human heart tissue with the assistance of UTMD. However, there are few reports on whether CMBs-mediated gene transfection can achieve targeted gene therapy in large-animal model of myocardial injury.

Therefore, in this study, we aimed to explore whether CMBs assisted by UTMD could achieve cardiac-targeted gene delivery in a porcine MI/R injury model and to evaluate whether co-administration of exogenous human SIRT3 (hSIRT3) and TIMP3 (hTIMP3) could exert myocardial protective functions. It is expected that this study would provide some theoretical and technical reference for gene therapy in cardiovascular diseases.

## 2 Materials and methods

All experimental procedures followed the Guidelines of the Care and Use of Laboratory Animals and were approved by the Ethics Committee for Animal Care and Use of the Second Affiliated Hospital of Harbin Medical University (No. KY2017- 050).

### 2.1 Groups

In this study, 48 Bama miniature pigs (male, 5-month-old, 25–30 kg) were included and randomly assigned to the Sham surgery group (n = 9) and a transthoracic surgery group to establish MI/R injury model (n = 39). Among the 39 MI/R injury model pigs, 15 did not receive any treatment (I/R group), 6 received CMBs carrying vectors (CMBs/Vector) followed by UTMD treatment (named as: I/R + Vector group), 6 received CMBs carrying hSIRT3 plasmid (CMBs/hSIRT3) followed by UTMD treatment (I/R + hSIRT3 group), 6 received CMBs carrying hTIMP3 (CMBs/hTIMP3) plasmid followed by UTMD treatment (I/R + hTIMP3 group). For dual-gene therapy group (I/R + hSIRT3+hTIMP3, n = 6), the subjects simultaneously received the CMBs/hSIRT3 and CMBs/hTIMP3 mixture at the dosages equivalent to those in respective monotherapy groups, followed by UTMD treatment.

### 2.2 Establishment of porcine MI/R injury models

Porcine MI/R injury was established by left anterior descending (LAD) coronary artery ligation for 45 min, followed by reperfusion, as previously described [29]. Briefly, anesthesia was induced using Zoletil (intramuscular injection, 5 mg/kg; Virbac, France), followed by orotracheal intubation with a mixture of 3 % isoflurane and oxygen to maintain surgical anesthesia [30]. Real-time arterial oxygen saturation, electrocardiography (ECG), heart rate, and blood pressure were continuously monitored during surgery. The heart and LAD artery were exposed after thoracotomy at the left Ⅳ/Ⅴ intercostal space. MI/R injury was induced by ligating the LAD with 4.0 polypropylene sutures at the second diagonal coronary branch for 45 min, followed by the release of ischemic sutures to achieve reperfusion. Myocardial color changes and ECG S-T segment elevation indicate successful ischemia. In Sham group, the sutures were crossed at the second diagonal coronary branch of the LAD artery without ligation. All the subjects received cefotaxime (intramuscular injection, 50 mg/kg) for 7 days to prevent infection.

### 2.3 Construction of CMBs

The CMBs were prepared using thin-film hydration and acoustic vibration as described in our previous study [31,32]. Briefly, 3-[N-(N, N-dimethylaminoethane)-carbamoyl] cholesterol hydrochloride (DC-Chol, Avanti, USA), 1,2-distearoyl-sn-glycerol-3-phosphoethanolamine phosphocholine (DPPC, Avanti, USA), and 1,2-distearoyl-sn-glycerol-3-phosphoethanolamine-N-[methoxy (polyethylene glycol)]-2000 (DSPE-PEG2000, Avanti, USA) were dissolved in chloroform in a mass ratio of 1:4:10. Next, the chloroform solution was placed in a rotary evaporator at 50 °C to volatilize the chloroform to obtain the lipid film. Subsequently, the lipid membranes were re-suspended with an appropriate amount of sterile 10 % glycerol (Sigma-Aldrich, USA), diluted with phosphate buffered saline (PBS), and dissolved at 42 °C for 30 min to obtain a uniform lipid solution, which was then divided into 1.5 mL sterile bottles. Finally, octafluoropropane gas (C_3_F_8_) was bubbled through the solution and vibrated for 60 s to obtain the gas-filled CMBs. The initial CMBs were diluted into a concentration of 1 × 10^9^ CMBs/mL, sterilized with ^60^Co-γ radiation, and stored at 4 °C until use. The surface potential, size distribution, and morphological characteristics were determined using a Zetasizer NANO ZS system (Malvern, UK) and transmission electron microscopy (TEM; JEOL, Japan).

### 2.4 Loading efficiency of exogenous hSIRT3 or hTIMP3 genes onto CMBs

The hSIRT3 plasmid encoding the hSIRT3 full-length open reading frame with EGFP tags (CMV-hSIRT3-EGFP-SV40-Kanamycin, GenePharma, China), hTIMP3 plasmid encoding the hTIMP3 full-length open reading frame with EGFP tags (CMV-hTIMP3-EGFP-SV40-Kanamycin, GenePharma, China), and corresponding negative empty plasmid with EGFP tags (CMV-EGFP-SV40-Kanamycin, GenePharma, China) were all independently encapsulated in *Escherichia coli (E. coli). E. coli* was cultured and amplified in Luria-Bertani medium, and the corresponding plasmids were extracted using an endotoxin-free plasmid extraction kit (TIANGEN, China) following the manufacturer's instructions.

The loading efficiency evaluation was performed as reported in literature [33,34]. Briefly, hSIRT3 or hTIMP3 plasmid with a mass of 10, 20, 40, 60, 80 μg were incubated with 5 × 10^8^ CMBs for 15 min at room temperature, followed by being centrifugated at 600 rpm for 5 min and separated into two layers: plasmid-bound CMBs appear in the upper layer, while the free plasmid in the lower layer. The lower phase was centrifuged at 12,000 rpm for 5 min with 0.45 μm filter to harvest the resuspended, unconjugated plasmid to determine the DNA concentration with the spectrophotometer (Thermo Fisher, USA). Therefore, the loading capacity of CMBs was defined as: (Total DNA - Free DNA)/CMBs number. And the loading gene encapsulation efficiency of CMBs was calculated as: [(Total DNA - Free DNA)/Total DNA] × 100 %. In addition, the efficiency of CMBs loading of hSIRT3 or hTIMP3 plasmids was analyzed by flow cytometry (BD Biosciences, USA) after CMBs/plasmids mixture being stained with propidium iodide (PI, Solarbio, China) at room temperature for 10 min.

### 2.5 CMBs/hSIRT3 and CMBs/hTIMP3 construction and characteristics

40 μg empty, hSIRT3, and hTIMP3 plasmids were incubated with 5 × 10^8^ CMBs respectively, at room temperature for 15 min to achieve electrostatic binding between CMBs and plasmids. After incubating the CMBs/Vector with PI solution (Solarbio, China) at room temperature for 10 min, the morphology and structure of the CMBs/Vector were observed under a fluorescence microscope (Leica, Germany). The surface potential, size distribution of the CMBs/hSIRT3 and CMBs/hTIMP3 were determined with Zetasizer NANO ZS system (Malvern, UK).

### 2.6 UTMD delivery in the porcine MI/R injury model

UTMD-mediated gene delivery in pigs was performed at the end of the surgery as described in our previous study [31]. Briefly, the subjects were maintained under anesthesia using a mixture of 3 % isoflurane and oxygen. The amount of plasmid (plasmid weight (mg) = animal weight (kg)/5) were calculated in advance based on previous studies [31]. Next, the empty, hSIRT3 and hTIMP3 plasmids were incubated with CMBs respectively at room temperature for 15 min and then transferred into 50 mL of physiological saline. For monogenic therapy group, the solution of CMBs/hSIRT3 (or CMBs/hTIMP3) were pumped into the circulatory system through the ear vein using a venous pump at 100 mL/h. And for dual-gene therapy group, the subjects were received the CMBs/hSIRT3 and CMBs/hTIMP3 mixture at the dosages equivalent to those in respective monotherapy groups. Simultaneously, the ultrasound beam was transmitted using an S5-1 transducer and the EPIQ5 system (Philips Medical Systems, Netherlands) with the probe frequency of 1.0–5.0 MHz and an ECG trigger for ultrasound burst at each fourth end-systole. The depth of the ultrasound beam was set at 9–13 cm. The mechanical index was set from 1.2 (low) to 1.35 (flash). The ultrasound beam continuously irradiated the long and short axial sections of the heart to burst the CMBs in the myocardium as fully as possible. The UTMD-mediated gene delivery was repeated three times at one-day intervals.

### 2.7 Echocardiography

Echocardiography was performed to evaluate cardiac function before and after gene therapy. An S5-1 transducer with a frequency of 1–5 MHz (Philips Medical Systems, Netherlands) was used to conduct transthoracic echocardiography. Short-axis images of the M-mode were collected, and left ventricular end systolic volume, end diastolic volume, left ventricular end systolic diameter and left ventricular end diastolic diameter were recorded and used to calculate the left ventricular ejection fraction (LV-EF) and fractional shortening rate (LV-FS) as described in our previous study [35].

### 2.8 Enzyme-linked immunosorbent assay (ELISA) assay

Serum cardiac troponin I (cTNI) levels were used to assess whether UTMD-mediated gene delivery induced myocardial injury. Venous blood samples were collected from the central vein of the pigs 24 h after the third UTMD treatment. The venous blood was centrifuged at 4 °C, 3000 rpm for 20 min and the supernatant serum was collected for ELISA assay following the instructions of a porcine cTNI ELISA kit (USCN Science, China). The absorbance was detected with the microplate reader at 450 nm (BioTek, USA).

### 2.9 Western blot assay

The protein expression levels of exogenous hSIRT3, hTIMP3, endogenous SIRT3, TIMP3, MnSOD, Catalase (CAT), MMP2, MMP9, caspase-3, B-cell lymphoma-2 (Bcl2) and Bcl2-associated X (Bax) were detected by western blot assay, as described in our previous study [31]. First, myocardial proteins were extracted using RIPA lysis solution (Beyotime, China) containing 1 mM PMSF (Beyotime, China), and their concentrations were detected using a BCA kit (Beyotime, China) according to the manufacturer's instructions. A total of 50 μg denatured proteins was loaded on the 10 % or 12.5 % SDS-PAGE gel for separation, followed by being transferred onto the PVDF membrane (Millipore, USA). The primary antibodies against hSIRT3 (1:1,000, ab217319, Abcam), hTIMP3 (1:1,000, ab276134, Abcam), SIRT3 (1:1,000, 10099-1-AP, Proteintech), TIMP3 (1:1,000, 10858-1-AP, Proteintech), MnSOD (1:5,000, 24127-1-AP, Proteintech), CAT (1:2,000, 21260-1-AP, Proteintech), MMP2 (1:500, 10373-2-AP, Proteintech), MMP9 (1:1,000, 10375-2-AP, Proteintech), caspase-3 (1:1,000, 66470-2-Ig, Proteintech), Bcl2 (1:1,000, ab32124, Abcam) and Bax (1:5,000, 50599-2-Ig, Proteintech) were incubated with the corresponding PVDF membranes overnight at 4 °C. Subsequently, the membranes were incubated with HRP-conjugated secondary antibody at room temperature for 1 h. Finally, the PVDF membranes were imaged using the ChemiDoc MP System (Bio-Rad, USA) using an ECL reagent (Beyotime, China) and quantitatively analyzed using ImageJ (Version 6.0).

### 2.10 RNA extraction, reverse transcription and polymerase chain reaction (PCR)

PCR was used to detect the expression levels of hSIRT3 and hTIMP3 in different organs, as described in our previous study [31]. Total RNA was extracted using TRIzol (Invitrogen, USA) following the manufacturer's instructions. Then, 1 μg RNA was used for reverse transcription with RT Easy II First Strand cDNA Synthesis Kit (Roche, Netherlands). The cDNA was amplified with the Mastercycler nexus PCR Detection System (Eppendorf, USA) according to thermocycling conditions: pre-heating at 95 °C for 3 min, followed by 27 cycles at 95 °C for 30 s, 60 °C for 30 s and 72 °C for 60 s. The primers used in the assay were as follows: hSIRT3 forward, CGGCTCTACACGCAGAACATCC; reverse, GCAGGTGGCAGAGGCAAAG; hTIMP3 forward, TCGGCACGCTGGTCTACAC; reverse, GTCAGCAGGTACTGGTACTTGTTG; and GAPDH forward, AGGCTGTGGGCAAGGTCATC; reverse, CTCCAGGCGGCAGGTCAG. Finally, the amplified samples were visualized on a 0.5 % agarose gel with a LightCyclerR480 II (Roche, Netherlands) and quantitatively analyzed using ImageJ (Version 6.0).

### 2.11 Terminal deoxynucleotidyl transferase-mediated dUTP nick-end labeling (TUNEL) staining assay

Apoptosis in the injured myocardium was evaluated by the TUNEL staining assay. Firstly, the myocardium was collected and fixed in 4 % paraformaldehyde (PFA) at 4 °C for 24 h. The fixed myocardium was then dehydrated in 10 %, 20 %, and 30 % sucrose solutions and embedded in optimal cutting temperature (OCT, SAKURA) embedding agent to yield 5-μm thick frozen slices with freezing microtome (Leica). Next, the TUNEL staining assay was performed with a TUNEL staining kit (Roche, Netherlands) following the manufacturer's protocol. After the slices were stained with 4′,6-diamidino-2-phenylindole (DAPI) solution at room temperature for 5 min, the images were obtained using a fluorescence microscope (Leica, Germany). The number of TUNEL-positive and DAPI-positive cells was analyzed using the ImageJ software (Version 6.0). The apoptosis level of the sample was calculated as the number of TUNEL-positive cells/DAPI-positive cells in five random fields.

### 2.12 Dihydroethidium (DHE) assay

The DHE assay was performed to detect the reactive oxygen species (ROS) level in injured myocardium. Firstly, the fresh myocardium was fixed and embedded in OCT agent to yield 5-μm thick frozen slices. Next, the DHE working solution (10 μM, Beyotime, China) was utilized to incubate the slices for 30 min in the dark at 37 °C. Finally, images were obtained with the fluorescence microscope (Leica, Germany) and analyzed for fluorescence intensity in five random fields with ImageJ (Version 6.0).

### 2.13 Immunohistochemistry (IHC) assay

The IHC assay was conducted for Cluster of differentiation 11b (CD11b), Collagen I (Col1a1), Interleukin-1β (IL-1β), Interleukin-6 (IL-6), and Tumor necrosis factor-α (TNF-α) expression detection in injured myocardium. Briefly, following the myocardium was fixed in 4 % PFA for 24 h, the myocardium was dehydrated in 75, 80, 90, and 100 % ethanol solution. After transparent treatment with xylene, the samples were embedded in paraffin to yield 4-μm thick sections using a paraffin slicer (Leica, Germany). After deparaffinization and rehydration, antigen retrieval was performed with sodium citrate buffer (10 mM) at 95 °C for 20 min. Endogenous peroxidase activity was quenched with 3 % hydrogen peroxide in methanol for 15 min at room temperature. After the non-specific binding sites being blocked with 5 % normal goat serum (Solarbio, China) for 1 h at room temperature, slices were then incubated overnight at 4 °C with primary antibodies against: CD11b (1:100, ab8878, Abcam), Col1a1 (1:100, ab138492, Abcam), IL-1β (1:100, ab2105, Abcam), IL-6 (1:100, ab216492, Abcam), and TNF-α (1:1000, ab307164, Abcam). Next, the slices were incubated with HRP-conjugated secondary antibody at room temperature for 1 h. Finally, slices were developed with DAB solution and examined under a microscope (Leica, Germany). To quantify IHC intensity, we used Image-Pro Plus (Version 6.0) to analyze the mean integrated optical density (mean IOD) value in five random fields.

### 2.14 Immunofluorescence (IF) assay

The IF assay was performed to detect the protein expression of Green fluorescent protein (GFP), CD11b, Cluster of differentiation 31 (CD31), and α-smooth muscle actin (α-SMA) in the injured myocardium as described in our previous study [31]. The myocardium had been fixed in 4 % PFA, dehydrated using a gradient sucrose solution, and embedded in OCT before being placed in the freezing microtome to obtain 5-μm thick frozen sections. Slices were firstly incubated with 0.5 % Triton X-100 solution for permeability at the room temperature for 30 min. Next, the slices were incubated with 5 % goat serum for 1 h at room temperature to exclude the influence of endogenous nonspecific protein antigens. Sequentially, the sections were incubated with pre-diluted primary antibodies against GFP (1:500, ab183734, Abcam), CD11b (1:100, ab8878, Abcam), CD31 (1:500, ab134168, Abcam), and α-SMA (1:1,000, ab7817, Abcam) at 4 °C overnight. The sections were then incubated with a fluorescent secondary antibody at room temperature for 1 h. After incubation with DAPI solution for 5 min at room temperature, the sections were observed and recorded under a fluorescence microscope (Leica, Germany). All protein expression was quantitatively analyzed in five random fields using ImageJ software (Version 6.0). GFP expression level was quantified as GFP-positive cells/DAPI-positive cells. The CD11b expression level was quantified as the number of CD11b-positive cells per unit area. CD31 and α-SMA expression levels were represented by the number of venous and arterial vessels per unit area.

### 2.15 Histological analysis

To evaluate the morphological changes in myocardial injury, we used Hematoxylin and Eosin (HE) staining to evaluate the pathological morphology of myocardial injury, whereas Masson and Sirius red (SR) staining was used to evaluate the deposition of collagen fibers in myocardial injury, as described in our previous study [31]. The fixed tissues underwent dehydration through graded ethanol solutions and were embedded in paraffin to obtain 4-μm thick sections. Next, HE, Masson's trichrome, and SR staining were performed via corresponding reagent kit according to manual instruction (Solarbio, China). Images were obtained using an optical microscope (Leica, Germany). ImageJ (Version 6.0) was used to quantify fibrosis/unit area in five random fields.

### 2.16 Evaluation of the size of myocardial scar area

The scar area was evaluated as previously described [36]. The hearts of each group were fully separated, cut into five equal parts from the apex of the heart to the bottom of the heart, and each part was separated by 1 cm. ImageJ (Version 6.0) was used to calculate the scar and total myocardial areas. The relative area of the scar was calculated as the total scar area/total myocardial area.

### 2.17 Statistical analysis

In this study, all experiments were repeated three or more times, and the experimental results are presented as the mean ± standard deviation (SD). Statistical analyses were performed and charts were produced using GraphPad Prism software (version 8.0). All the schematic diagrams and flowcharts showed in the study were performed on the online tool of Figdraw from HOME for Researchers (https://www.home-for-researchers.com/static/index.html#/). The statistical comparison between two groups was performed using an unpaired *t*-test, and the comparison between three or more groups was performed using one-way ANOVA, followed by Tukey's multiple comparison test. Statistical significance was set at *P* < 0.05.

## 3 Results

### 3.1 Establishment of porcine MI/R injury model

**Fig. 1:**
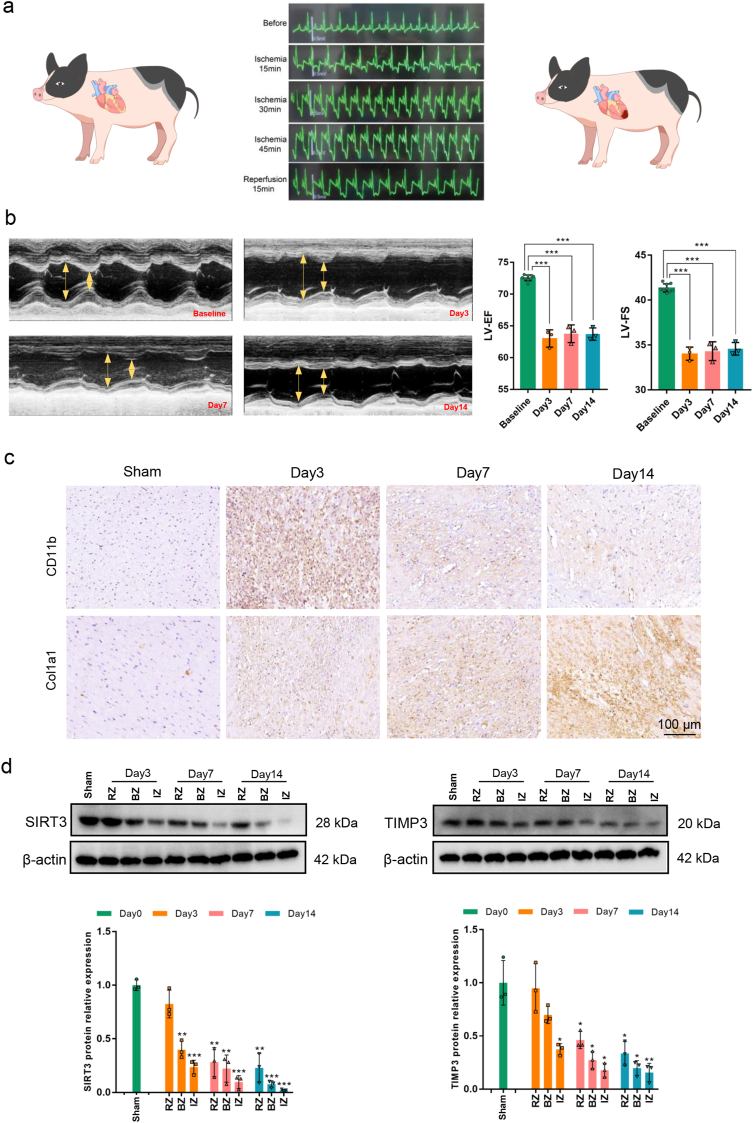
**Characteristics of myocardial and cardiac function in a porcine MI/R injury model. a.** Dynamic ECG in the establishment of porcine MI/R injury model. **b.** the level of LVEF, LVFS at baseline and days 3, 7, 14 after MI/R injury. **c.** Representative images for CD11b and Col1a1 expressions in IZ myocardium of the Sham group and on days 3, 7, and 14 after MI/R injury (scale bar = 100 μm). **d.** The protein expressions and quantitative analysis of endogenous SIRT3 and TIMP3 in IZ, BZ, and RZ in the Sham group and on days 3, 7, and 14 after MI/R injury (∗*P* < 0.05, ∗∗*P* < 0.01, ∗∗∗*P* < 0.001 vs. Sham group). Data are presented as the Means ± SD (n = 3). ECG: Electrocardiogram. LV-EF: Left ventricular ejection fraction. LV-FS: Left ventricular fractional shortening. IZ: Infarction zone. BZ: Border zone. RZ: Remote zone.

First, the porcine MI/R injury model was verified using ECG monitoring. Before LAD ligation, no abnormal changes were observed on the ECG. After 15 min of ischemia, the S-T segment of the ECG was depressed, and the T-wave was inverted. After 30 min and 45 min of ischemia, the progression of S-T depression and T-wave inversion over time became clearer. However, after restoration of coronary blood flow, the level of the S-T segment recovered, and the T-wave was upright (Fig. 1a). We further examined the cardiac function on days 3, 7, and 14 after surgery. Before surgery the baseline LV-EF was 72.57 ± 0.45 % and LV-FS was 41.35 ± 0.46 %. On days 3, 7, and 14 after surgery, the LV-EF values were 63.02 ± 1.36 %, 63.77 ± 1.39 %, 63.71 ± 0.97 %, respectively, and the LV-FS values were 34.03 ± 0.73 %, 34.31 ± 1.05 %, 34.58 ± 0.70 %, respectively. The LV-EF and LV-FS were significantly lower on days 3, 7, and 14 after operation compared to baseline (*P* < 0.001); however, there was no significant difference among the levels on days 3, 7, and 14 (*P* > 0.05, Fig. 1b). These results indicate that MI/R injury can cause a decrease in cardiac function compared with baseline of healthy heart, but in the short period after injury, there is no trend of worsening or worsening of cardiac function.

### 3.2 The pathological and molecular changes in porcine MI/R injury

HE staining was performed to evaluate the pathomorphology of the myocardium after MI/R injury. As shown in Fig. S1, compared with the normal myocardium of the Sham group, the infiltration of inflammatory cells was the highest on day 3 and gradually decreased, with myocardial cell swinging, destruction, and death. On day 14 after MI/R injury, collagen fibers were observed in the area of myocardial injury. In addition, we selected CD11b and Col1a1, the biomarkers for a variety of inflammatory/immune cells and collagen deposition in extracellular matrix, to validate the inflammatory cells infiltration and myocardial fibrosis levels after myocardial injury. Consistent with HE assays, the CD11b expression was highest on the day 3 and decreased on day 7 and day 14 thereafter. Col1a1 expression gradually increased after myocardial injury, with the highest expression observed in the tissue on day 14 (Fig. 1c). The result aligns with previous findings indicating that the acute inflammatory phase of myocardial injury primarily occurs within the first 3 days post-injury, followed by a decline in inflammation during days 3–7 as the tissue transitions to the proliferative phase, characterized by fibroblast proliferation and ECM deposition [37,38]. For molecular perspective, in the infarct zone (IZ) myocardium, both SIRT3 and TIMP3 proteins were significantly decreased on day 3 after MI/R injury compared to the Sham group (*P* < 0.05), and their expression continued to decrease on days 7 and 14, with the lowest expression at day 14. In the border zone (BZ) and remote zone (RZ) myocardium, the expression levels of SIRT3 and TIMP3 gradually decreased over time. Compared with their expression levels in the BZ and RZ, the expression of SIRT3 and TIMP3 proteins decreased most significantly in the IZ (Fig. 1d). These results suggest that even if the ischemic myocardium undergoes revascularization, there are still biological processes that cannot be ignored, such as inflammatory reactions, cell apoptosis, and myocardial fibrosis. Simultaneously, whether in the early acute inflammation phase or in the subsequent proliferation and maturation phases, the expression of SIRT3 and TIMP3 continues to decrease, especially in the IZ myocardium, suggesting SIRT3 and TIMP3 may be the crucial regulators involved in the myocardial repair post-injury. This phenomenon inspired us to investigate whether rescuing the expression of SIRT3 and TIMP3 in the early stage of MI/R injury can exert a protective effect on myocardial injury.

### 3.3 Characterization of CMBs/hSIRT3 and CMBs/hTIMP3

CMBs is an ideal vector for targeted delivery of exogenous genes or drugs. And if CMBs mediated gene therapy can play a myocardial protective role in the porcine, it would have major significance for the future clinical transformation of CMBs mediated gene therapy.

**Fig. 2:**
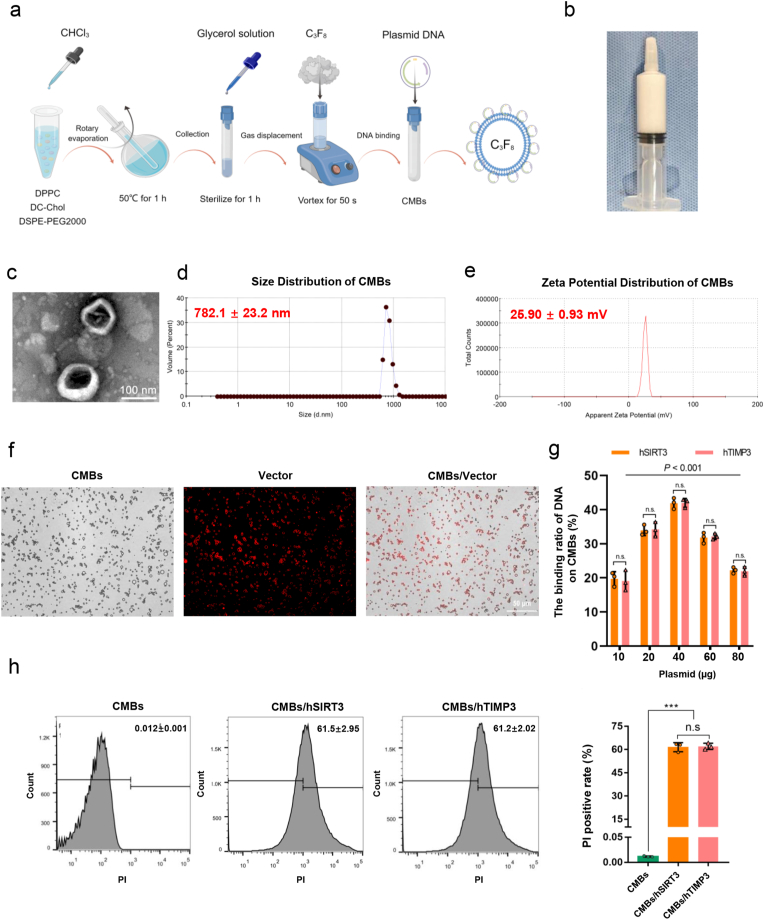
**Characteristics of CMBs. a.** Schematic diagram of CMB preparation. **b.** The appearance of CMBs. **c.** Microscopic morphology of CMBs detected by TEM (scale bar = 100 nm). **d.** The distributions of diameter and **(e)** surface charge of CMBs. **f.** The morphology of CMBs/vector labeled with PI stain (red). Scale bar = 50 μm. **g.** The encapsulation efficiency of CMBs for different doses of hSIRT3 or hTIMP3 genes. **h.** The loading rate of CMBs with hSIRT3 and hTIMP3 plasmids detected by flow cytometry. Data are presented as the Means ± SD (n = 3). CMBs: Cationic microbubbles. TEM: Transmission electron microscopy. hSIRT3: human SIRT3. hTIMP3: human TIMP3. ∗∗∗*P* < 0.001. n.s.: not significant. (For interpretation of the references to color in this figure legend, the reader is referred to the Web version of this article.)

Firstly, CMBs were prepared as shown in Fig. 2a. Macroscopically, the CMBs appeared as a milky white suspension (Fig. 2b) and a membrane-coated hollow spherical structure under TEM condition (Fig. 2c). The diameter and surface charge of CMBs were 782.1 ± 23.2 nm (Figs. 2d), 25.90 ± 0.93 mV (Fig. 2e), respectively. Next, the morphology of the CMBs/Vector was observed using a fluorescence microscope. As shown in Fig. 2f, the CMBs/Vector retained a regular circular shape, and the empty plasmid as displayed by PI was attached to the surface of the CMBs. It was determined that CMBs at unit concentration achieved their maximum binding capacity with 40 μg of plasmid (Fig. 2g). At this condition, the encapsulation efficiency of CMBs for the hSIRT3 or hTIMP3 genes was determined as 41.9 ± 1.3 % and 42.9 ± 1.1 % respectively, without statistical difference (*P* > 0.05). And the gene loading capacity of CMBs for hSIRT3 or hTIMP3 genes was 33.5 ± 1.1 μg/10^9^ CMBs, 33.6 ± 0.8 μg/10^9^ CMBs, respectively, without statistical difference (*P* > 0.05). And the average loading rates of CMBs with hSIRT3 and hTIMP3 plasmids analyzed by flow cytometry were 61.5 ± 2.95 % and 61.2 ± 2.02 %, respectively. And these were not statistically different on loading rate between CMBs/hSIRT3 and CMBs/hTIMP3 (Fig. 2h, *P* > 0.05). These data demonstrated a consistent capability of CMBs to load both the hSIRT3 or hTIMP3 genes. The diameters and surface charges of the CMBs/hSIRT3 and CMBs/hTIMP3 were also measured. The results revealed that CMBs/hSIRT3 diameter and surface charge were 936.0 ± 85.2 nm and −0.68 ± 2.21 mV, respectively (Fig. S2a and b), and the CMBs/hTIMP3 diameter and surface charge were 910.0 ± 63.5 nm and −1.60 ± 0.64 mV, respectively (Fig. S2c and d). The changes in these characteristics indicated that the CMBs effectively loaded the plasmid and reached saturation.

### 3.4 Cardiac-targeted delivery of CMBs/hSIRT3 and CMBs/hTIMP3 by UTMD

**Fig. 3:**
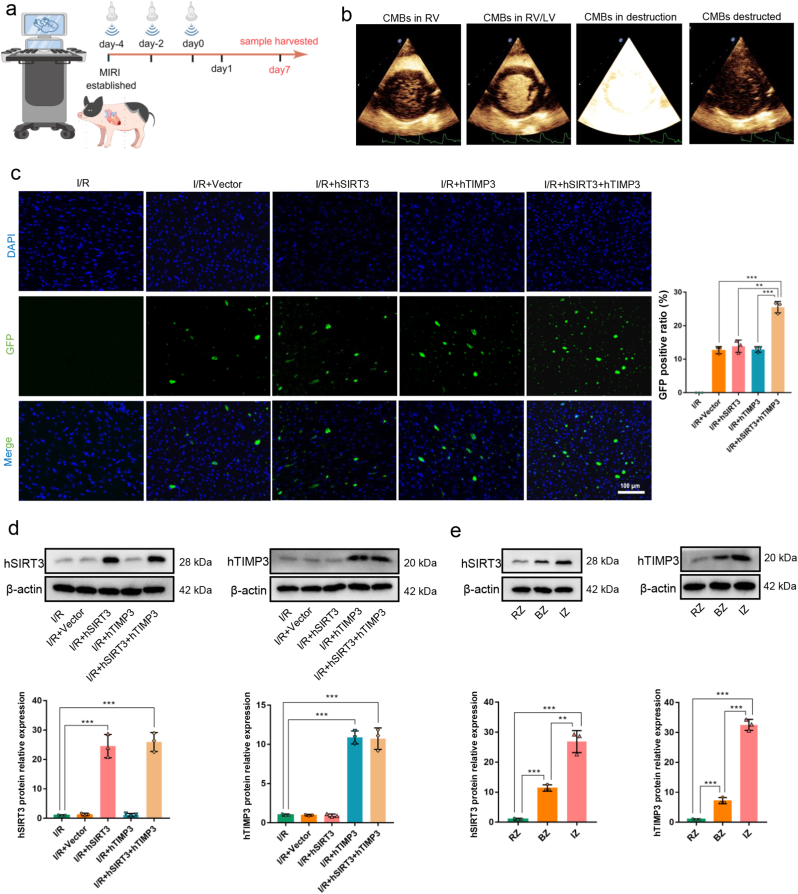
**Cardiac-targeted delivery of hSIRT3 and hITMP3 plasmids by UTMD. a.** Schematic diagram of UTMD procedure for cardiac-targeted delivery of hSIRT3 and hITMP3 plasmids. **b.** Representative ultrasound contrast images of imaging and destructing CMBs/hSIRT3 or CMBs/hTIMP3 in the heart. **c.** Representative images of GFP expression in the myocardium and quantitative analysis of GPF expression among the groups. **d.** Protein expressions and quantitative analysis of hSIRT3 and hTIMP3 among the groups. **e.** Protein expressions and quantitative analysis of hSIRT3 and hTIMP3 among RZ, BZ and IZ myocardium. Data are presented as the Means ± SD (n = 3). UTMD: Ultrasound-targeted microbubble destruction. CMBs: Cationic microbubbles. hSIRT3: human SIRT3. hTIMP3: human TIMP3. IZ: Infarction zone. BZ: Border zone. RZ: Remote zone. ∗∗*P* < 0.01, ∗∗∗*P* < 0.001.

The time and observation frequency of the objects subjected to the UTMD processing are shown in Fig. 3a. Representative images of the process of CMBs carrying plasmids refluxing into the right heart through the venous system and perfusing the whole heart, followed by the destruction and release of plasmids are shown in Fig. 3b. After 7 days of observation, we detected the expression level of GFP protein in the IZ region to test whether the plasmids loaded on CMBs effectively entered the myocardium. The results revealed that compared with the I/R group without UTMD treatment, GFP was clearly expressed in the groups that received UTMD treatment, including the I/R + Vector, I/R + hSIRT3, I/R + hTIMP3, and I/R + hSIRT3+hTIMP3 groups (Fig. 3c). In I/R + hSIRT3+hTIMP3 group, due to the subjects simultaneously received the CMBs/hSIRT3 and CMBs/hTIMP3 mixture at the dosages equivalent to those in respective monotherapy (I/R + hSIRT3 or I/R + hTIMP3) groups and GFP from hSIRT3 and hTIMP3 plasmids both expressed in the myocardium, resulting the GFP expression in I/R + hSIRT3+hTIMP3 group was significantly higher than other monotherapy groups (*P* < 0.001, Fig. 3c).

Furthermore, the protein expression levels of hSIRT3 were the same between the I/R + hSIRT3 and I/R + hSIRT3+hTIMP3 groups (*P* > 0.05) and was significantly higher than that in the other groups (*P* < 0.001, Fig. 3d). Similarly, hTIMP3 protein expression levels between the I/R + hTIMP3 and I/R + hSIRT3+hTIMP3 groups showed no statistical difference (*P* > 0.05) and were significantly higher than that in the other groups (*P* < 0.001, Fig. 3d), suggesting in the dual-gene therapy group, co-administration of CMBs/hSIRT3 and CMBs/hTIMP3 mixture ensured equivalent per-gene transfection efficiency compared to their corresponding monotherapy groups. Notably, after UTMD treatment, the protein expressions of hSIRT3 and hTIMP3 were mainly concentrated in the IZ myocardium, and was significantly higher than that in the BZ and RZ myocardium (*P* < 0.001, Fig. 3e). These results suggest that UTMD effectively delivered the exogenous gene into the myocardium, and the IZ myocardium with the most severe injury received more gene transfection.

### 3.5 Safety and targeting evaluation of UTMD-mediated gene delivery

**Fig. 4:**
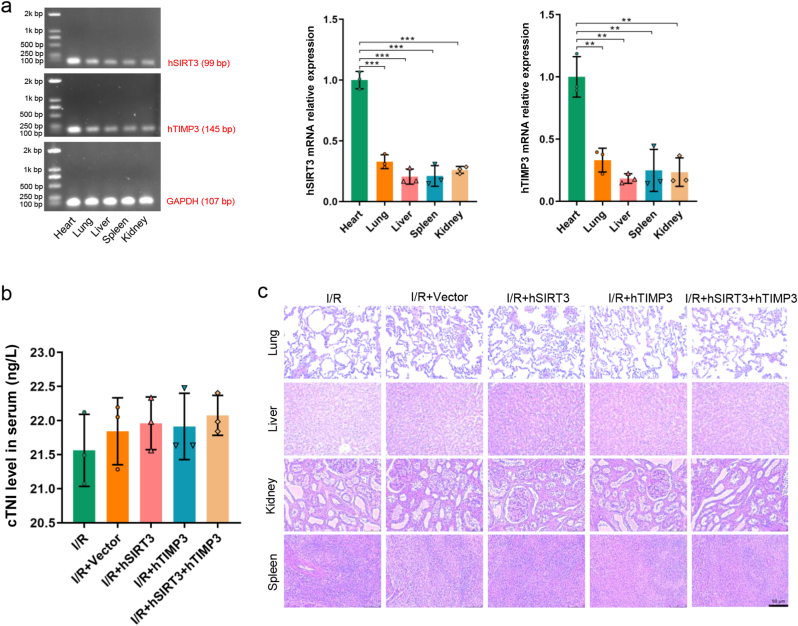
**Safety and targeting evaluation of UTMD-mediated gene delivery. a.** hSIRT3 and hTIMP3 gene expressions and quantitative analysis in the lungs, liver, spleen, and kidneys. **b.** Comparison of serum cTNI levels among different groups. **c.** Morphology images of the lungs, liver, spleen, and kidneys of different groups (scale bar = 50 μm). Data are presented as the Means ± SD (n = 3). UTMD: Ultrasound-targeted microbubble destruction. ∗∗*P* < 0.01, ∗∗∗*P* < 0.001.

We detected the expression levels of hSIRT3 and hTIMP3 in different organs to evaluate the targeting of UTMD-mediated gene delivery. As shown in Fig. 4a, the gene expression of hSIRT3 and hTIMP3 were significantly enriched in the heart than in other organs (*P* < 0.01). hSIRT3 and hTIMP3 were also expressed in a small amount in organs other than the heart, and we hypothesized that a small number of hSIRT3 and hTIMP3 plasmids circulated in blood after ultrasound destruction, resulting in low gene expression in other organs. Consistent with expectations, there was no significantly difference in serum cTNI levels between subjects receiving UTMD treatment and those without UTMD treatment (*P* > 0.05, Fig. 4b). In addition, compared with the subjects without UTMD treatment, the groups with UTMD treatment did not exhibit clear pathological manifestations such as bleeding, inflammatory infiltration, and necrosis in the lungs, liver, kidneys, and spleen (Fig. 4c). The above results indicate that UTMD-mediated targeted transfection of the porcine injury heart ensured transfection efficiency without causing clear damage to the myocardium and other important organs.

### 3.6 Short-term evaluation of targeted exogenous hSIRT3 and hTIMP3 gene myocardial therapy

**Fig. 5:**
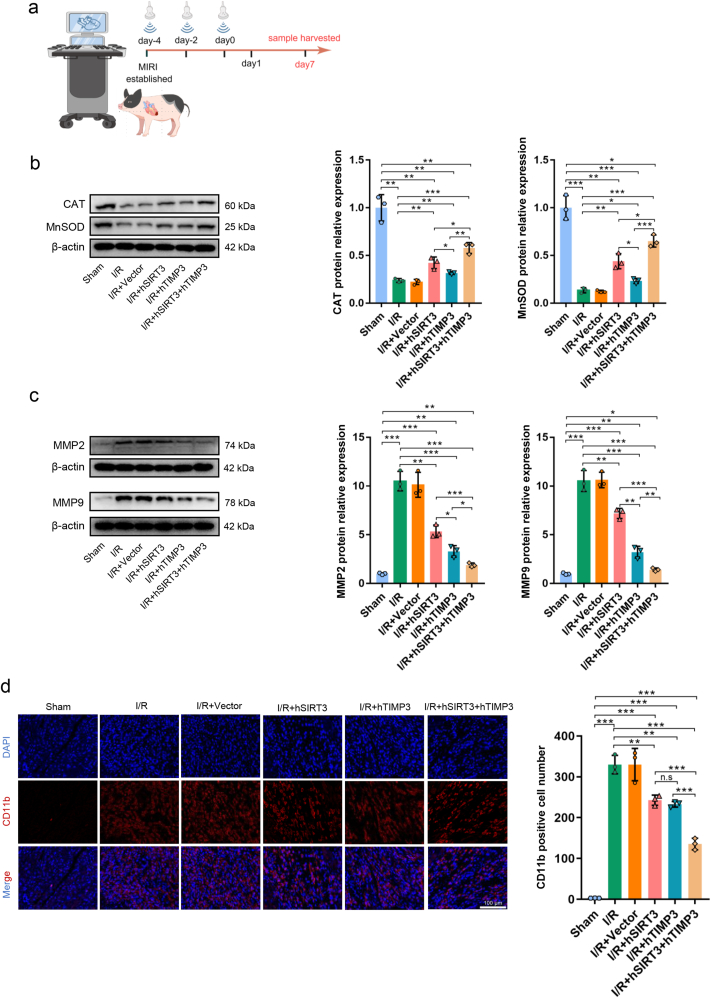
**Exogenous hSIRT3 and hTIMP3 genes exerted their downstream signaling regulation and inhibited the infiltration of inflammatory cells. a.** Flowchart of short-term effects of exogenous hSIRT3 and hTIMP3 genes on myocardial injury. **b.** Protein expressions and quantitative analysis of CAT and MnSOD among the different groups. **c.** Protein expressions and quantitative analysis of MMP2 and MMP9 among the different groups. **d.** Representative images of CD11b expression in the myocardium and quantitative analysis of CD11b expression among the different groups (scale bar = 100 μm). Data are presented as the Means ± SD (n = 3). hSIRT3: human SIRT3. hTIMP3: human TIMP3. ∗*P* < 0.05, ∗∗*P* < 0.01, ∗∗∗*P* < 0.001. n.s.: not significant.

The subjects with UTMD were observed for 7 days after receiving different gene therapies, and the short-term effects of gene therapy were evaluated (Fig. 5a). To evaluate whether exogenous hSIRT3 and hTIMP3 genes in the myocardium could exert myocardial protective effects, we first detected downstream proteins of SIRT3 and TIMP3 in the IZ myocardium. As expected, both hSIRT3 and hTIMP3 effectively increased the expression levels of CAT and MnSOD (*P* < 0.05); however, hSIRT3 had a significantly higher promoting effect than hTIMP3 (*P* < 0.05). Simultaneously, the synergistic effect of hSIRT3 and hTIMP3 in the I/R + hSIRT3+hTIMP3 group showed a higher promoting effect than that in the hSIRT3 or hTIMP3 monotherapy therapy (*P* < 0.05, Fig. 5b). The elevation of antioxidant enzymes is contributed to alleviate the myocardial ROS level. As expected, the ROS level in IZ myocardium was significantly decreased after receiving exogenous gene therapy (*P* < 0.01). And the synergistic application of hSIRT3 and hTIMP3 could significantly inhibit ROS compared to hSIRT3 or hTIMP3 individual application (*P* < 0.05). Meanwhile, the inhibitory effect of hTIMP3 on ROS was weaker than that of hSIRT3 (*P* < 0.05, Fig. S3). These results indicate that although hSIRT3 is more effective than hTIMP3 in enhancing the antioxidant capacity of myocardium, their combined application could exert a greater protective effect against myocardial oxidative stress injury. For downstream molecules of TIMP3, both hSIRT3 and hTIMP3 effectively inhibited the expressions of MMP2 and MMP9 (*P* < 0.01). hTIMP3 had a stronger inhibitory effect on MMP2 and MMP9 than hSIRT3 (*P* < 0.05), and the expression levels of MMP2 and MMP9 were lowest in the I/R + hSIRT3+hTIMP3 group compared to the I/R + hSIRT3 or I/R + hTIMP3 groups (*P* < 0.05, Fig. 5c). This phenomenon indicates that hSIRT3 could assist hTIMP3 in further inhibiting the expression of MMP2 and MMP9.

**Fig. 6:**
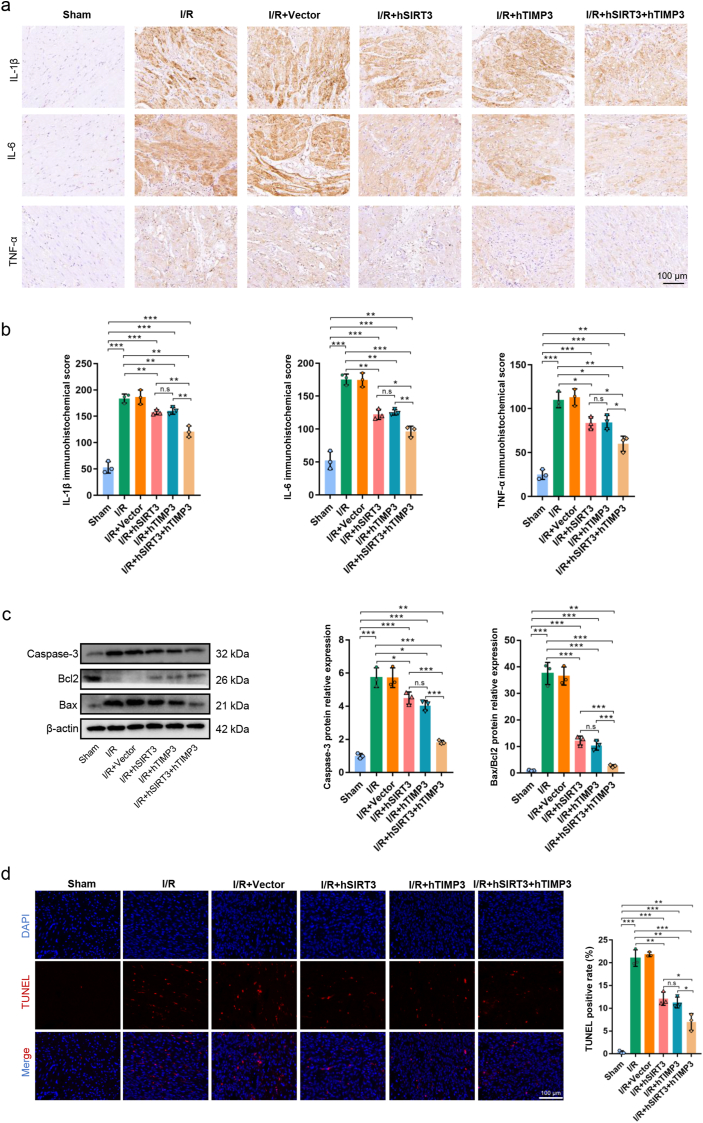
**Exogenous hSIRT3 and hTIMP3 genes inhibited apoptosis of myocardium. a.** Representative images of IL-1β, IL-6 and TNF-α expressions in IZ myocardium and quantitative analysis **(b)** among the different groups (scale bar = 100 μm). **c.** Protein expressions and quantitative analysis of caspase-3, Bcl2, and Bax among the different groups. **d.** Representative images of TUNEL-positive cells in the myocardium and quantitative analysis of TUNEL-positive cells among the different groups (scale bar = 100 μm). Data are presented as the Means ± SD (n = 3). hSIRT3: human SIRT3. hTIMP3: human TIMP3. ∗*P* < 0.05, ∗∗*P* < 0.01, ∗∗∗*P* < 0.001. n.s.: not significant.

Myocardial injury is accompanied by excessive inflammatory reactions, and the inhibition of the inflammatory process during myocardial injury can effectively inhibit ventricular remodeling and fibrosis, delaying the progression of HF [39]. In the IZ myocardium, the number of CD11b-positive cells significantly decreased in the I/R + hSIRT3 and I/R + hTIMP3 groups (*P* < 0.01); however, no statistical difference was observed between the two monotherapy groups (*P* > 0.05). In the I/R + hSIRT3+hTIMP3 group, the number of CD11b-positive cells further decreased compared to that in hSIRT3 or hTIMP3 monotherapy groups (*P* < 0.001, Fig. 5d). In addition, we also detected the IL-1β, IL-6 and TNF-α expressions in the IZ myocardium. As expected, although gene therapy application all significantly suppressed the levels of inflammatory cytokines, the synergistic application of hSIRT3 and hTIMP3 could significantly inhibit the expressions of IL-1β, IL-6, and TNF-α compared to using hSIRT3 or hTIMP3 alone (*P* < 0.001). And there was no significant difference observed between the use of hSIRT3 or hTIMP3 alone (*P* > 0.05, Fig. 6a and b).

Saving cardiomyocytes at the early stage of myocardial injury is conducive to maintaining the number of functional cells and delaying the decline in cardiac function [30]. Apoptosis related proteins in the IZ were compared among the groups. As expected, compared to the I/R group, application of hSIRT3 or hTIMP3 genes exhibited the same inhibitory effect on the expression of apoptosis-related index of caspase-3 and Bax/Bcl2 (*P* < 0.05), and there was no statistical difference between the I/R + hSIRT3 and I/R + hTIMP3 groups (*P* > 0.05). Similarly, the combined effect of hSIRT3 and hTIMP3 exhibited a more significant inhibitory effect on apoptosis-related proteins than the single-gene therapy (*P* < 0.001, Fig. 6c). The anti-apoptotic effects of hSIRT3 and hTIMP3 on myocardial injury were also confirmed by the TUNEL staining assay, which revealed that hSIRT3 and hTIMP3 had a similar ability to inhibit apoptosis and that their synergistic effect was more significant (Fig. 6d).

These results indicate that exogenous hSIRT3 and hTIMP3 effectively regulate downstream signaling molecules in the myocardium. hSIRT3 and hTIMP3 effectively inhibited inflammatory level and tissue apoptosis in the early period of myocardial injury, and the synergistic effect of dual-gene treatment was more significant than that of single-gene treatment.

### 3.7 Long-term evaluation of exogenous hSIRT3 and hTIMP3 gene targeted myocardial therapy

**Fig. 7:**
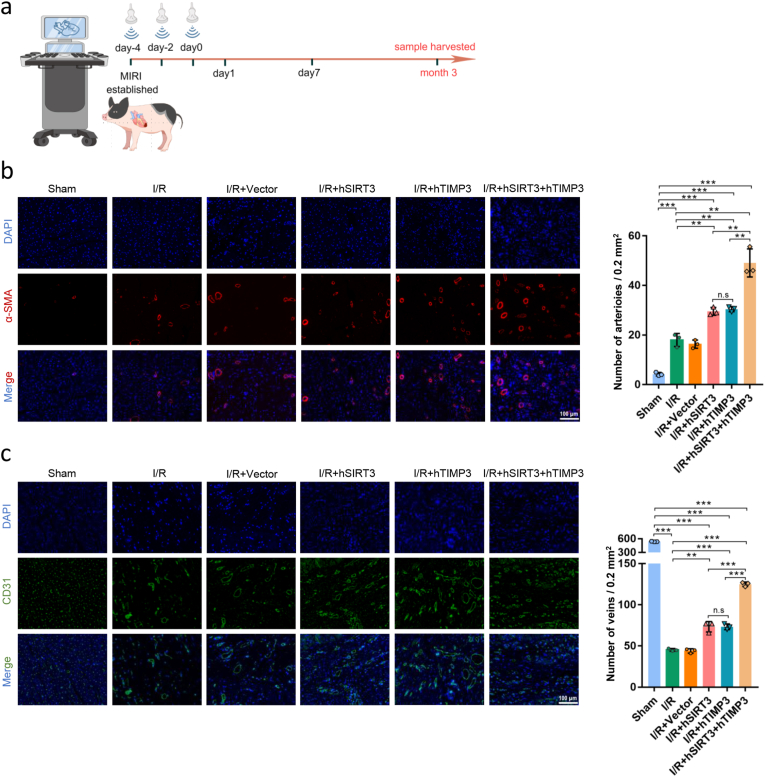
**Exogenous hSIRT3 and hTIMP3 genes promoted revascularization after myocardial injury. a.** Flowchart of long-term effects of exogenous hSIRT3 and hTIMP3 genes on myocardial injury. **b.** Representative images of α-SMA labeled arteries and **(c)** CD31 labeled veins in the myocardium and quantitative analysis among the different groups (scale bar = 100 μm). Data are presented as the Means ± SD (n = 3). hSIRT3: human SIRT3. hTIMP3: human TIMP3. ∗∗*P* < 0.01, ∗∗∗*P* < 0.001. n.s.: not significant.

The subjects were observed for 90 days after receiving different gene therapies with UTMD, and the long-term effects of gene therapy were evaluated (Fig. 7a). Locally abundant revascularization after myocardial injury is associated with better cardiac function [30]. We labeled arterial and venous vessels with α-SMA and CD31 molecules, respectively, to evaluate vascular remodeling in the IZ myocardium after MI/R injury. The results suggest that after hSIRT3 or hTIMP3 gene therapy, the arterial and venous vessels in the I/R + hSIRT3 and I/R + hTIMP3 groups were significantly improved compared to those in the I/R group (*P* < 0.01). However, there was no significant difference between hSIRT3 and hTIMP3 in promoting vascular reconstruction (*P* > 0.05), and the synergistic effect of dual-gene therapy in the I/R + hSIRT3+hTIMP3 group promoted vascular reconstruction better (*P* < 0.01) than that in the I/R + hSIRT3 or I/R + hTIMP3 group (Fig. 7b and c).

**Fig. 8:**
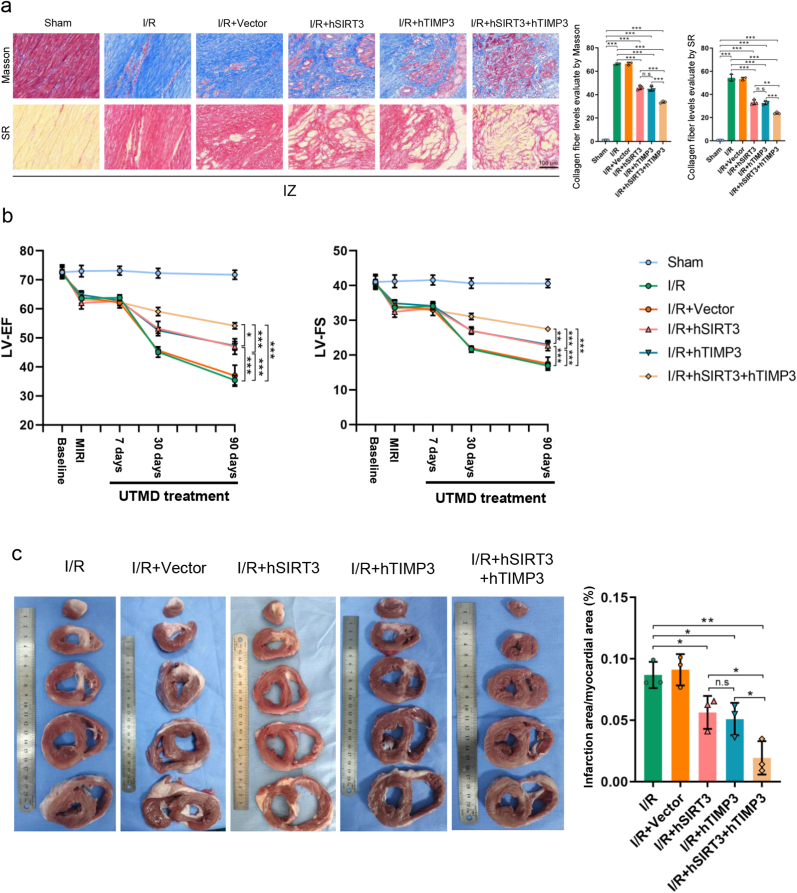
**Exogenous hSIRT3 and hTIMP3 genes suppressed myocardial fibrosis after myocardial injury. a.** Representative images of collagen deposition and myocardial fibrosis of IZ stained by Masson's and SR assays and quantitative analysis among the different groups (scale bar = 100 μm). **b.** Dynamic values of LV-EF, LV-FS at baseline and different time points after gene therapy among the groups. **c.** Representative images and quantitative analysis of fibrotic scar among the different groups. Data are presented as the Means ± SD (n = 3). hSIRT3: human SIRT3. hTIMP3: human TIMP3. IZ: Infarction zone. BZ: Border zone. RZ: Remote zone. LV-EF: Left ventricular ejection fraction. LV-FS: Left ventricular fractional shortening. ∗*P* < 0.05, ∗∗*P* < 0.01, ∗∗∗*P* < 0.001. n.s.: not significant.

Important characteristics of adverse ventricular remodeling are collagen deposition and fibrosis in the myocardial tissue. We performed Masson's and SR staining of the myocardium in the IZ, BZ, and RZ myocardium after MI/R injury to evaluate whether hSIRT3 and hTIMP3 gene therapy could improve adverse ventricular remodeling. As expected, with hSIRT3 or hTIMP3 alone therapy, the collagen fiber deposition levels in the I/R + hSIRT3 and I/R + hTIMP3 groups were significantly decreased compared to those in the I/R groups, in all regions (*P* < 0.01). Simultaneously, with hSIRT3 and hTIMP3 cooperation treatment, the collagen fiber deposition level in the I/R + hSIRT3+hTIMP3 group was further decreased compared to that in the I/R + hSIRT3 or I/R + hTIMP3 groups (*P* < 0.01, Fig. 8a and Fig. S4).

We evaluated cardiac function on days 7, 30, and 90 after gene therapy. As expected, on day 7 after gene therapy, there was no significant difference among the different groups. However, on day 30, LV-EF and LV-FS values in the hSIRT3 or hTIMP3 gene monotherapy groups were significantly higher than those in the I/R group. LV-EF and LV-FS in the hSIRT3 and hTIMP3 synergistical therapy group were further increased compared with those in the hSIRT3 or hTIMP3 gene monotherapy group. After 90 days of observation, LV-EF and LV-FS in the I/R + hSIRT3+hTIMP3 group were significantly higher than those in the I/R + hSIRT3 and I/R + hTIMP3 groups (Fig. 8b and Fig. S5). Lastly, through observation of whether individual or synergistic application, hSIRT3 and hTIMP3 gene therapy could effectively limit the size of the scar area after MI/R injury, as expected, synergistic therapy showed better myocardial protection than monotherapy (Fig. 8c).

These results indicate that individual or synergistic application of hSIRT3 and hTIMP3 gene can promote local revascularization, inhibit collagen deposition and fibrosis, and limit scar size, thereby preserving cardiac function and delaying the progression of HF after MI/R injury of pig.

## 4 Discussion

To some extent, MI/R injury weakens the expected benefits of coronary revascularization for ischemic heart disease. Previous studies have confirmed that some drugs, such as statins (atorvastatin) [3], adenosine [5], erythrocyte-stimulating hormone [4], and mitochondrial function protection drugs [40] could be used for cardioprotection against MI/R injury. However, these strategies have not achieved promising results in some clinical trials. The reasons for failure of clinical outcomes are diverse, and the most important factor is the selection of experimental animals. Based on the experimental conclusions of rodents, owing to the significant differences in gene expression regulation and physiological metabolism compared to humans, the relevance to the pathogenesis or treatment of human diseases is relatively poor [36]. Therefore, we used pigs with physiological and anatomical functions similar to those of the human cardiovascular system as experimental subjects to explore the protective effect of gene-targeted therapy based on UTMD technology on MI/R injury to provide meaningful theoretical and technical references for improving the prevention and treatment of MI/R injury.

A significant advantage of gene therapy is that it can inhibit pathogenic gene, restore favorable gene expression to achieve disease treatment [41]. It has been gradually applied in clinical practice and has become an important means of treating genetic diseases, infectious diseases, and tumors [42]. In a clinical trial for HF gene therapy, an adenovirus carrying the sarcoplasmic/endoplasmic reticulum calcium ion ATPase 2a (SERCA2a) gene was directly injected into the coronary arteries to enhance the myocardial calcium ion processing capacity and improve cardiac pump function. However, the final results revealed no significant differences in New York Heart Association's heart function grading, heart failure-related readmission events, and the 6-min walking test between patients receiving gene therapy and those receiving placebo [43,44]. This indicates that a precise target is a prerequisite for successful gene therapy. In our study, the protein expression levels of endogenous SIRT3 and TIMP3 decreased over time in the IZ, BZ, and RZ myocardium, particularly in the IZ. SIRT3 and TIMP3 have been confirmed in multiple reports, including our previous study, to exert a role in myocardial protection by regulating biological processes such as oxidative stress and ECM remodeling post-MI [17,[45], [46], [47]]. There have been reported that injecting the exogenous TIMP3 gene directly into the edge of myocardial infarction effectively limited scar enlargement, adverse ECM remodeling, and cardiac function decline [18]. Further studies are needed to determine whether TIMP3, as a target for gene therapy, has a superior myocardial protective effect compared to other gene targets, such as SIRT3, and whether TIMP3 and SIRT3 combination therapies could work synergistically for myocardial protection.

The principle of UTMD-mediated gene transfection is that when microbubbles are blasted using an ultrasonic beam, they can produce cavitation- and voice-controlled effects on local endothelial cells, thus widening their gaps and increasing capillary permeability [48,49]. Simultaneously, with the help of the driving force generated by microbubble blasting, various genes or drugs can cross the vascular barrier and enter tissue cells [50]. In the present study, CMBs loaded with hSIRT3 and hTIMP3 were used for cardiac-targeted gene delivery. hSIRT3 and hTIMP3 proteins were mainly enriched in the IZ myocardium with the most severe myocardial damage. After myocardial injury, the vascular endothelium is damaged, leading to widening of the endothelial space [51], which is more conducive for UTMD-mediated gene transfection. This may be the main reason for the significant enrichment of hSIRT3 and hTIMP3 proteins in the IZ myocardium. Moreover, cardiac-targeted gene delivery by UTMD did not cause additional or pathological damage to the myocardial tissue or other organs. These results indicate that UTMD-mediated cardiac-targeted gene delivery can effectively concentrate exogenous genes in the myocardial injury area.

Numerous studies have confirmed that SIRT3 plays a protective role against myocardial injury, including antioxidant stress damage, the promotion of angiogenesis, and the inhibition of myocardial cell apoptosis [[52], [53], [54], [55]]. SIRT3 also inhibits the expressions of MMP family members. In our previous study, we found that increased SIRT3 expression in the myocardium of a porcine model of pathological myocardial hypertrophy effectively inhibited the expression of MMP2 and MMP9, limiting the progression of pathological myocardial hypertrophy [31]. Similarly, TIMP3 had a regulatory effect on apoptosis, angiogenesis, and ECM remodeling of myocardial infarction [[56], [57], [58], [59]]. However, TIMP3 overexpression can alleviate oxidative stress and inflammation in metabolic disorders [56,60]. In an *in vivo* and *in vitro* study on diabetic retinopathy, a decrease in TIMP3 expression was accompanied by an increase in oxidative stress injury, which was manifested by an increase in ROS and a decrease in SOD and CAT enzyme activities. Changing the expression of TIMP3 in the eyeball by exogenous means can effectively increase SOD and CAT levels [61]. Consistent with the above reports, our results showed that the levels of downstream MnSOD and CAT proteins were significantly increased, and a decreased ROS level after the administration of hSIRT3 in the injured myocardium. Furthermore, hTIMP3 application significantly inhibited the levels of MMP2 and MMP9 proteins in myocardial tissue. In addition, we observed that hSIRT3 had an inhibitory effect on MMP2 and MMP9, and hTIMP3 also promoted an increase in MnSOD, CAT protein and inhibited ROS levels. These results indicate that hSIRT3 and hTIMP3 have a synergistic effect on oxidative injured and MMPs regulation.

Accordingly, to evaluate whether hSIRT3 and hTIMP3 have therapeutic effects on MI/R injury in pig, we observed the short- and long-term protective effects of hSIRT3 and hTIMP3 on myocardial injury on day 7 and 90 after gene transfection, respectively. After 7 days of treatment, hSIRT3 and hTIMP3 showed significant inhibitory effects on myocardial apoptosis and inflammatory levels. Similarly, after three months of observation, the groups receiving gene therapy showed significant advantages in terms of arteriovenous vascular density, myocardial fibrosis, scar size, and cardiac function. As expected, the simultaneous administration of hSIRT3 and hTIMP3 showed better protective effects than the administration of hSIRT3 or hTIMP3 alone. This result indicates that the simultaneous application of hSIRT3 and hTIMP3 had synergistic effects on cardiac protection.

Notably, for both short- and long-term observation indicators, no significant differences were observed between the groups receiving hSIRT3 or hTIMP3 gene therapy alone. We believe that the biological processes of myocardial injury, such as inflammation, apoptosis, fibrosis, and angiogenesis, are regulated by a variety of factors [62,63]. The regulatory ability of individual factors on these complex biological activities is limited, and it may not be possible to determine which factor is stronger or weaker than another single factor. However, the two factors responsible for these different functions would definitely collaborate with each other to a certain extent, thus playing an enhanced therapeutic role [64]. In addition, the dual-gene therapy strategy has been recognized to improve the pathological state of cells and to have a better therapeutic effect [65]. Overexpression of SERCA1 and Kir2.1 genes in the myocardium has been reported to effectively shorten the myocardial refractory period without limiting myocardial contractility, corresponding to the functions of SERCA1 and Kir2.1 genes, respectively. HF rats treated with SERCA1 and Kir2.1 genes had a significantly improved cardiac function [66]. This study was similar to our findings that hSIRT3 and hTIMP3 exert synergistic myocardial protective effects against MI/R injury. In terms of mechanism, the coordination and interaction between hSIRT3 and hTIMP3 on inflammation-related molecules and signaling are crucial for their synergistic effect in protecting the injured myocardium. As reported, abnormal expression of SIRT3 can lead to reduced levels of antioxidant enzymes such as SOD in cardiomyocytes, resulting in oxidative stress damage to the cells. Subsequently, oxidative stress triggers inflammatory responses via the nuclear factor kappa-B (NF-κB) signaling pathway, inducing compensatory hypertrophy of cardiomyocytes, vascular dysfunction and excessive collagen and fibronectin deposition. These pathological alterations collectively drive ventricular remodeling, ultimately progressing to HF [47,67]. At the same time, TIMP3 is revealed that it could alleviate inflammation by suppressing the NF-κB signaling pathway [67]. Specifically, it inhibits nuclear translocation of NF-κB, thereby downregulating the expressions of its downstream factors [68]. And in our results, levels of inflammatory cytokines (IL-1β, IL-6, and TNF-α) were significantly reduced in both individual hSIRT3 or hTIMP3 treatment groups, with the most pronounced reduction observed in the combination therapy group, suggesting hSIRT3 and hTIMP3 may exert their synergistic effects through inflammation related NF-κB signaling mechanisms.

Our study had some limitations. First, there were only 3 subjects in each group. With an increase in sample size, there may be some errors in the statistical analysis of certain results. For animal transfections, different transfection dose groups should be established to determine the most appropriate transfection dose. The stability of plasmid-loaded CMBs in systemic circulation was not systematically assessed. In theory, higher stability would correlate with improved targeted transfection efficiency. Echocardiography applied in the study had some limitations compared with nuclear magnetic resonance in assessing cardiac function. In addition, the continuous refinement of CMBs surface charge and lipid composition ratios, improvement of cationic materials, stability of CMBs in the circulation and the exploration of additional sonication parameters to achieve more efficient targeted delivery of exogenous genes remain crucial research in UTMD-mediated targeted gene transfection.

## 5 Conclusion

**Fig. 9:**
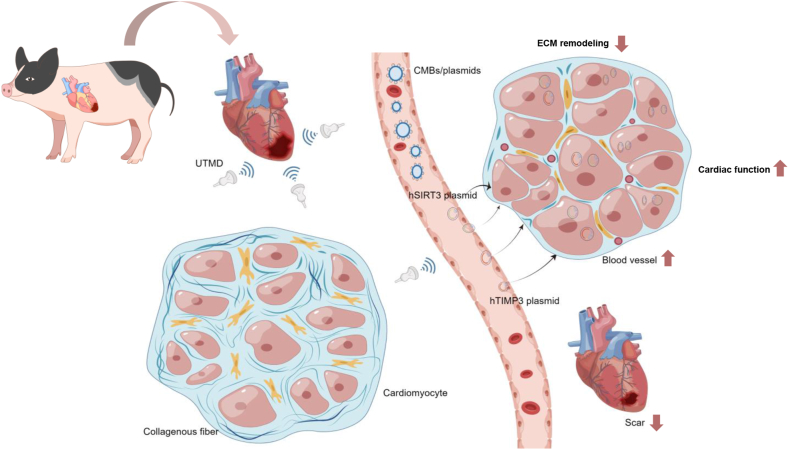
**Schematic illustration of UTMD-mediated protective effects of exogenous hSIRT3 and hTIMP3 genes in a porcine MI/R injury model.** UTMD effectively delivers exogenous hSIRT3 or hTIMP3 genes to the damaged myocardium via CMBs carriers, which suppresses early-stage myocardial inflammation and apoptosis, limits ECM remodeling, and promotes angiogenesis and cardiac functional recovery. Meanwhile, the combination of hSIRT3 and hTIMP3 genes can better exert myocardial protective effects compared to their individual application.

With the assistance of UTMD technology, CMBs achieved targeted delivery of exogenous genes into the myocardium of a porcine MI/R injury model. hSIRT3 or hTIMP3 genes could suppress early-stage myocardial inflammation and apoptosis, limits ECM remodeling, and promotes angiogenesis and cardiac functional recovery. The synergistic application of hSIRT3 and hTIMP3 genes demonstrated significantly enhanced therapeutic outcomes compared to monogenic therapy (Fig. 9). Our study provides advanced technical insights into UTMD-mediated *in vivo* targeted gene therapy, while validating the myocardial protective effects of SIRT3 and TIMP3 genes in a porcine model. These findings offer critical foundations for further clinical translation of this strategy.

**Table:** Abbreviations

Bax	B-cell lymphoma-2-associated X
Bcl2	B-cell lymphoma 2
BZ	Border zone
C_3_F_8_	Octafluoropropane gas
CAT	Catalase
CD11b	Cluster of differentiation 11b
CD31	Cluster of Differentiation 31
CMBs	Cationic microbubbles
CMBs/Vector	CMBs loaded with vector plasmid
CMBs/hSIRT3	CMBs loaded with hSIRT3 plasmid
CMBs/hTIMP3	CMBs loaded with hTIMP3 plasmid
Col1a1	Collagen I
cTNI	Cardiac troponin i
DAPI	4′,6-diamidino-2-phenylindole
DC-Chol	3-[N-(N, N-dimethylaminoethane)-carbamoyl] cholesterol hydrochloride
DHE	Dihydroethidium
DPPC	1,2-distearoyl-sn-glycerol-3-phosphoethanolamine phosphocholine
DSPE-PEG2000	1,2-distearoyl-sn-glycerol-3-phosphoethanolamine-N-[methoxy (polyethylene glycol)]-2000
ECG	Electrocardiography
ECM	Extracellular matrix
ELISA	Enzyme-linked immunosorbent assay
GFP	Green fluorescent protein
HE	Hematoxylin and Eosin
HF	Heart failure
hSIRT3	Human sirtuin 3
hTIMP3	Human tissue inhibitors of metalloproteinases 3
IF	Immunofluorescence
IHC	Immunohistochemistry
IL-1β	Interleukin-1β
IL-6	Interleukin-6
IZ	Infarct zone
LAD	Left anterior descending
LV-EF	Left ventricular ejection fraction
LV-FS	Left ventricular fractional shortening rate
MI	Myocardial infarction
MI/R	Myocardial ischemia-reperfusion
MMPs	Matrix metalloproteinases
MnSOD	Manganese superoxide dismutase
NF-κB	Nuclear factor kappa-B
OCT	Optimal cutting temperature
PBS	Phosphate buffered saline
PCR	Polymerase chain reaction
PFA	Paraformaldehyde
PI	Propidium iodide
ROS	Reactive oxygen species
RZ	Remote zone
SD	Standard deviation
SERCA2a	Sarcoplasmic/endoplasmic reticulum calcium ion atpase 2a
SIRT3	Sirtuin 3
SR	Sirius red
TEM	Transmission electron microscopy
TIMP3	Tissue inhibitors of metalloproteinases 3
TNF-α	Tumor necrosis factor-α
TUNEL	Terminal deoxynucleotidyl transferase-mediated dutp nick-end labeling
UTMD	Ultrasound-targeted microbubble destruction
α-SMA	α-smooth muscle actin

## CRediT authorship contribution statement

**Peian Cai:** Writing – review & editing, Writing – original draft, Project administration, Methodology, Formal analysis, Data curation, Conceptualization. **Kegong Chen:** Writing – review & editing, Project administration, Methodology, Investigation, Formal analysis, Data curation. **Xionghai Qin:** Validation, Project administration, Methodology, Formal analysis, Data curation. **Xingpei Jiang:** Visualization, Validation, Software, Data curation. **Xuan Jiao:** Visualization, Validation, Supervision, Methodology. **Kexun Liu:** Supervision, Resources, Methodology, Formal analysis. **Erliang Guo:** Validation, Supervision, Software. **Zipeng Li:** Validation, Supervision. **Xianxin Qiu:** Writing – review & editing, Supervision. **Chang Liu:** Project administration, Methodology, Conceptualization. **Lu Sun:** Resources, Project administration, Methodology, Conceptualization. **Junbo Chuai:** Software, Project administration. **Jie Wu:** Supervision, Resources, Methodology, Investigation, Conceptualization. **Wei Chen:** Writing – review & editing, Validation, Supervision, Methodology, Conceptualization. **Hai Tian:** Supervision, Project administration, Investigation, Funding acquisition, Conceptualization.

## Declaration of competing interest

The authors declare that they have no known competing financial interests or personal relationships that could have appeared to influence the work reported in this paper.

## Data Availability

Data will be made available on request.
