# Supplementary material for: Cationic microbubble loading hSIRT3 and hTIMP3 optimize cardiac-targeted delivery and myocardial protection in the porcine MI/R model
Source: Mater Today Bio. 2025 Aug 22;34:102234. doi: 10.1016/j.mtbio.2025.102234 (PMC12398931; doi:10.1016/j.mtbio.2025.102234)
Supplement: Multimedia component 1 [file mmc1.docx]

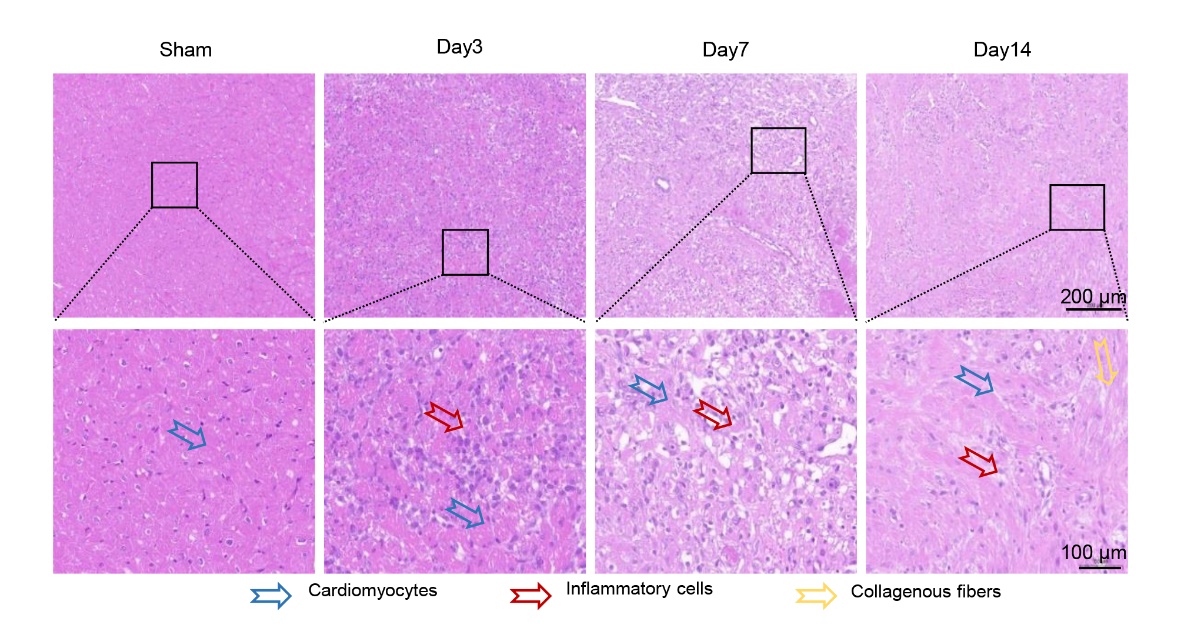


**Figure S1. Pathological characteristics of myocardium in the porcine MI/R injury model.** Representative images for myocardium in the Sham surgery group and on days 3, 7, and 14 after MI/R injury (scale bar = 200 µm (Up) and 100 µm (Below)). Blue arrow: Cardiomyocyte. Red arrow: Inflammatory cell. Yellow arrow: Collagen fibers.


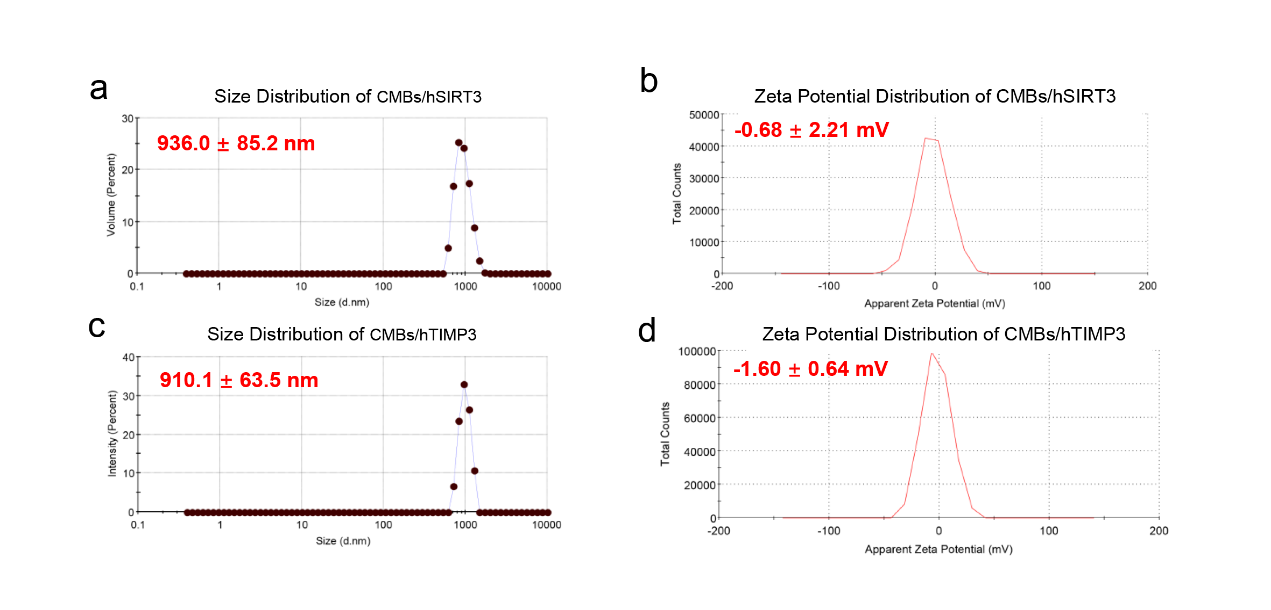
**Figure S2: Characteristics of CMBs/hSIRT3 and CMBs/hTIMP3. a.** The distributions of diameter of CMBs/hSIRT3. **b.** The surface charge of CMBs/hSIRT3. **c.** The distributions of diameter of CMBs/hTIMP3. **d.** The surface charge of CMBs/hTIMP3. CMBs: Cationic microbubbles. hSIRT3: human SIRT3. hTIMP3: human TIMP3.


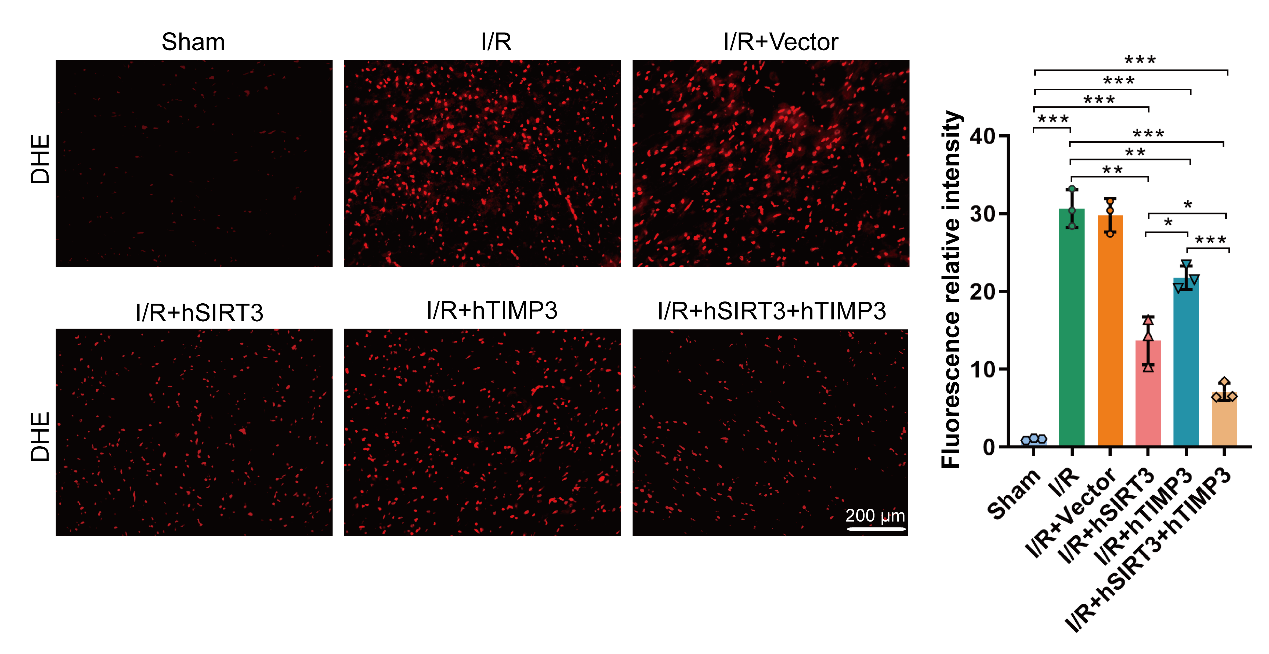


**Figure S3: Exogenous hSIRT3 and hTIMP3 genes alleviated the ROS level after myocardial injury.** Representative images of ROS level in IZ myocardium and quantitative analysis among the different groups (scale bar = 200 µm). Data are presented as the Means ± SD (n = 3). hSIRT3: human SIRT3. hTIMP3: human TIMP3. ROS: Reactive oxygen species. IZ: Infarction zone. * *P* < 0.05, ** *P* < 0.01, *** *P* < 0.001.


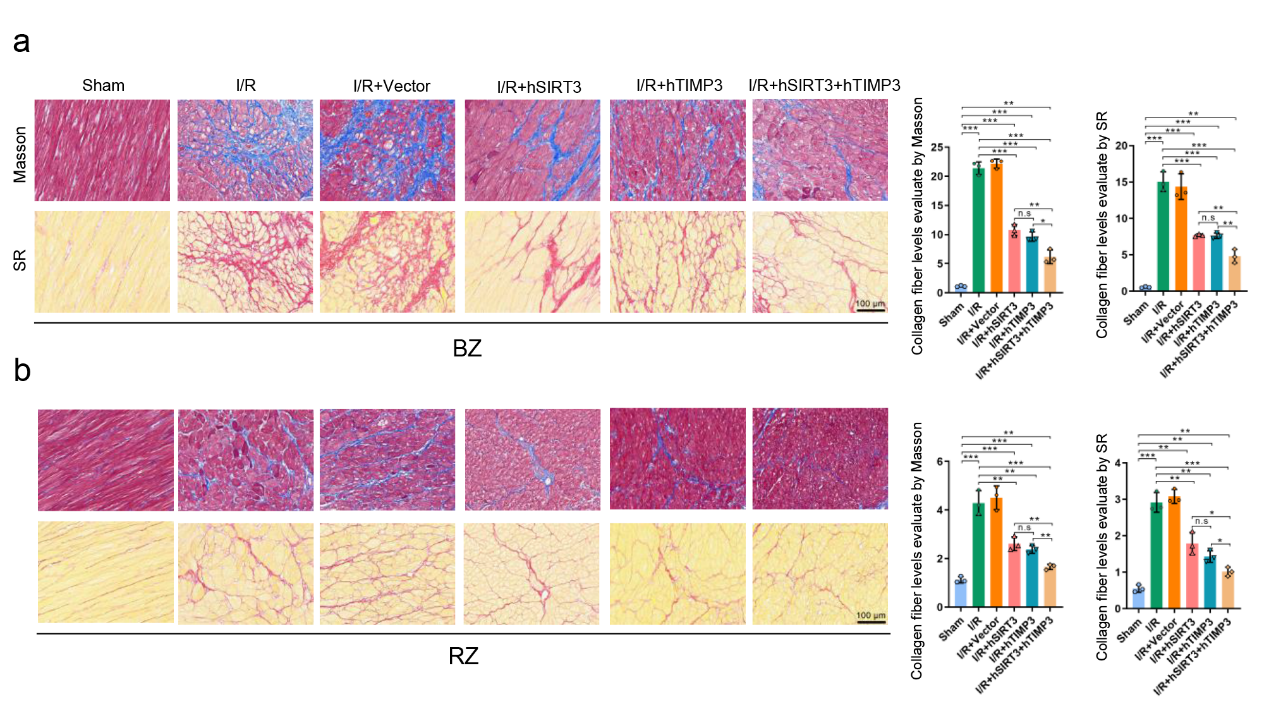


**Figure S4: The level of collagen deposition and myocardial fibrosis in BZ and RZ a.** Representative images of collagen deposition and myocardial fibrosis of BZ, and **(b)** RZ stained by Masson’s and SR assays and quantitative analysis among the different groups (scale bar = 100 µm). Data are presented as the Means ± SD (n = 3). BZ: Border zone. RZ: Remote zone. * *P* < 0.05, ** *P* < 0.01, *** *P* < 0.001. n.s.: not significant.


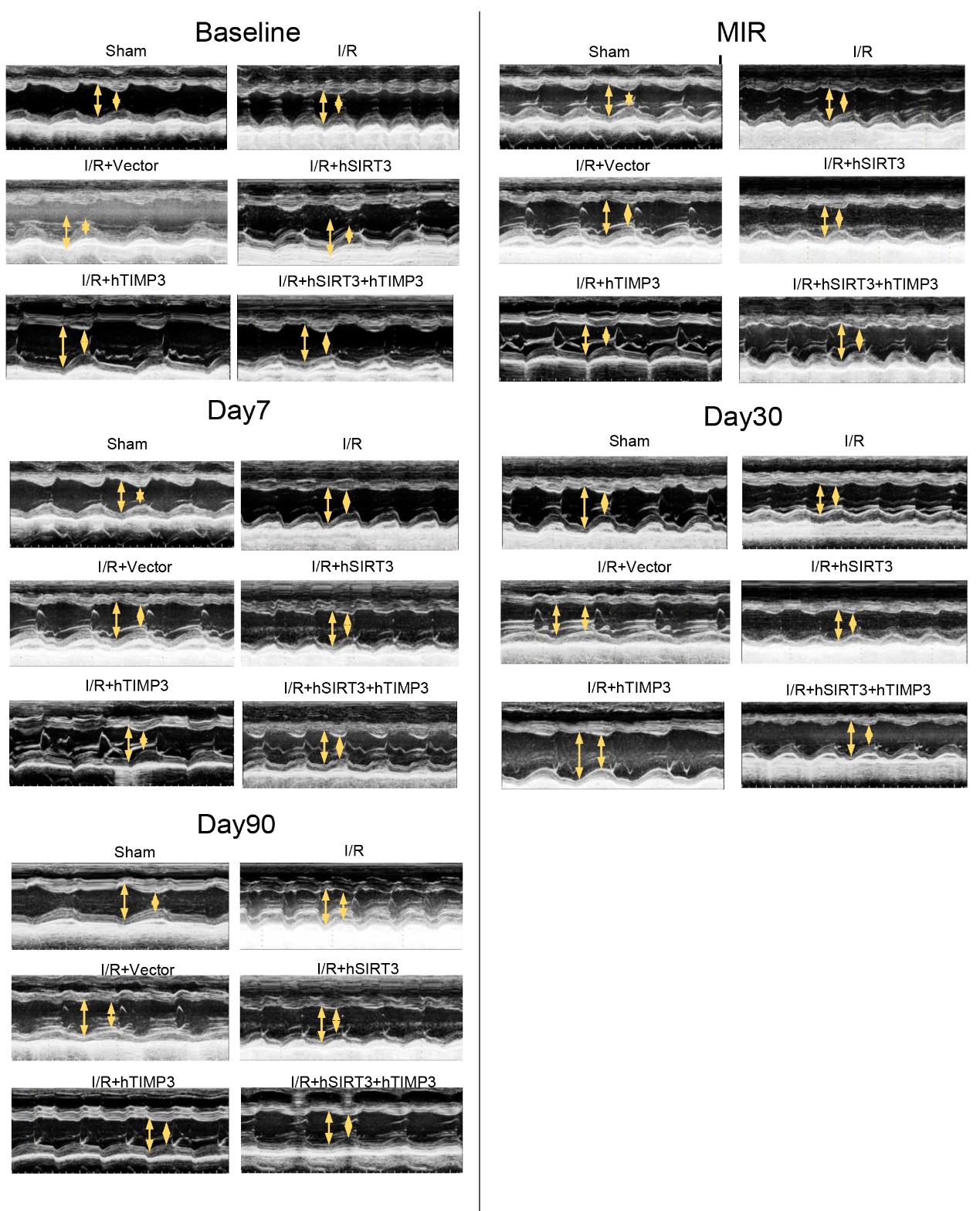


**Figure S5: The represent short-axis images of** **M-mode on the baseline, MI/R model establishment and on the 7th, 30th, and 90th day after receiving different treatment.**
